# Stress-induced reverse martensitic transformation in a Ti-51Ni (at%) alloy aged under uniaxial stress

**DOI:** 10.1038/s41598-018-24411-1

**Published:** 2018-04-17

**Authors:** Fei Xiao, Hong Chen, Xuejun Jin, Zhihua Nie, Tomoyuki Kakeshita, Takashi Fukuda

**Affiliations:** 1 State Key Lab of Metal Matrix Composite, School of Materials Science and Engineering, Shanghai Jiao Tong University, 800 Dong Chuan Road, Shanghai, 200240 P. R. China; 2 Institute of Advanced Steels and Materials, School of Materials Science and Engineering, Shanghai Jiao Tong University, Shanghai, 200240 People’s Republic of China; 3 School of Materials Science and Engineering, Beijing Institute of Technology, Beijing, 100081 China; 4 Department of Materials Science and Engineering, Graduate School of Engineering, Osaka University, 2-1, Yamada-oka, Suita, Osaka, 565-0871 Japan

## Abstract

Ti-51Ni (at%) alloys including coherent precipitates of Ti_3_Ni_4_ exhibits thermally-induced B2-R transformation. If the Ti_3_Ni_4_ is formed under tensile stress, it orientates preferentially so that its habit plane becomes perpendicular to the tensile axis. In such specimens, stress-induced reverse R-B2 transformation is reported to occur. In the present study, the stress-induced reverse R-B2 transformation behavior was studied by infrared camera and *in situ* X-ray analysis. The infrared camera observation revealed that the temperature of the specimen decreases homogeneously by the application of tensile stress within the resolution of the camera. The *in situ* X-ray analysis revealed that stress-induced reverse R-B2 transformation and rearrangement of variants of the R-phase occurs simultaneously in the specimen.

## Introduction

Martensitic transformations (MTs) are diffusionless structural changes^1^. They are first order transformations associated with a jump in extensive variables, such as entropy, volume, magnetization and strain^2,3^. Because of the jump in extensive variables, MTs can be controlled by changing the conjugate intensive variables, such as temperature, pressure, magnetic field and stress^3–5^. Among these intensive variables, temperature is different from the other variables because it is not a general force, while the other variables are regarded as general forces, which supply energy to the system though work, not through heat. These intensive variables (pressure, magnetic field, and stress) are frequently referred to as external fields.

Application of pressure stabilizes the martensite phase when the specific volume of the martensite is lower than that of the parent phase. This behavior can be found in Ni-Mn-Ga shape memory alloys (SMAs)^6^ and Cu-based SMAs^7^. On the other hand, it stabilizes the parent phase in Fe-Ni alloys^8^. Pressure-induced MT occurs in the former case, while pressure-induced reverse MT occurs in the latter case.

Application of magnetic field stabilizes the martensite phase when the magnetization of the martensite phase is larger than that of the parent phase. This behavior can be found in Fe-Ni and other iron based alloys^9,10^. On the other hand, it stabilizes the parent phase in Ni-Mn-In-(Co), Ni-Mn-Sn and Fe-Rh-Pd alloys^11–13^. Magnetic field-induced MT occurs in the former case while magnetic field-induced reverse MT occurs in the latter case.

As described above, both hydrostatic pressure and magnetic field stabilize the martensite phase in some alloy systems while they stabilize the parent phase in some other alloy systems. Application of a uniaxial stress, however, is frequently interpreted to stabilize only the martensite phase^14–16^. This implies that a stress-induced MT is possible, but a stress-induced reverse MT is impossible. This interpretation arises because there is always at least one variant of the martensite phase which can be induced with an orientation favorable to the direction of the externally applied stress (e.g. extending in the tensile direction or contracting in the compressive direction). In contradiction to such interpretation, a stress-induced reverse MT is possible when a strong internal stress is formed in the specimen that selects a specific variant of the martensite phase^17,18^.

A stress-induced reverse MT was observed in a Ni-rich Ti-Ni alloy aged under a tensile stress. The aged specimen includes coherent particles of Ti_3_Ni_4_ precipitate and transforms from the B2-phase to the R-phase in the cooling process. These particles are preferentially oriented so that the habit plane normal of Ti_3_Ni_4_ becomes nearly perpendicular to the tensile axis^17^. The coherent particles exert an internal stress field around the surrounding matrix. The internal stress field formed by the aligned Ti_3_Ni_4_ plays the key role for the stress-induced reverse transformation. The R-phase formed in the B2-matrix is composed of four variants. The aligned particles of Ti_3_Ni_4_ select a specific variant of the R-phase; we call this variant V1 and the other variants V2. (We regard that V2 is composed of three variants of the R-phase in this paper). The variant V1 is contracted to the tensile axis and extended to the direction perpendicular to the tensile axis. On the other hand, the variant V2 is extended to the tensile axis; therefore, V1 is unstable under external tensile stress, and it transforms into the B2-phase or V2.

**Figure 1:**
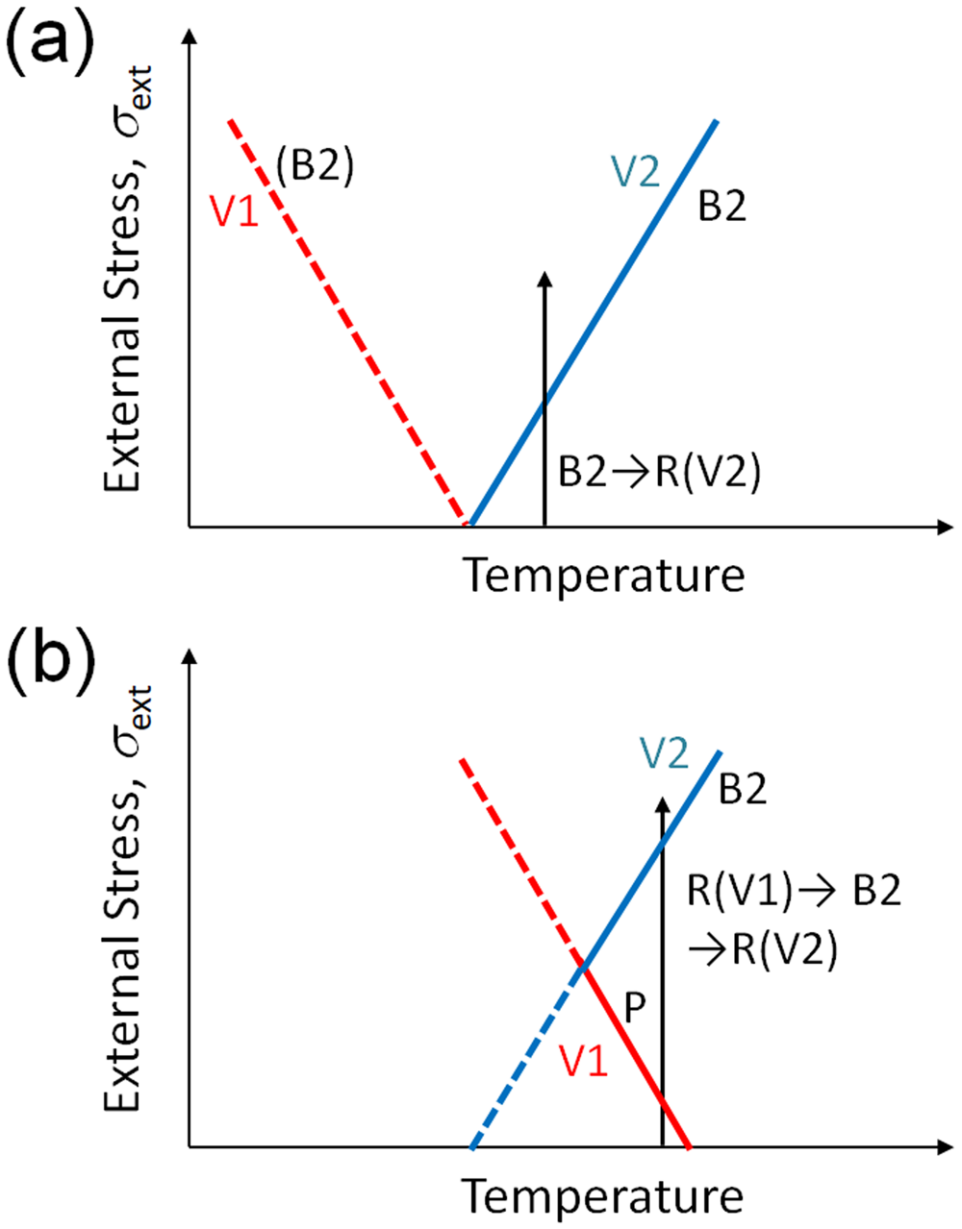
Schematic illustration showing the roles of internal stress and external stress on stress-induced martensitic transformation in the Ti-Ni SMAs: (**a**) without internal stress; (**b**) with internal stress formed by aligned particles of Ti_3_Ni_4_.

We propose that the role of internal stress and external stress on stress-induced transformation can be described by the phase diagram illustrated in Fig. 1. In general, the influence of stress, *σ*, on the transformation temperature, *T*, is given by the Clausius-Clapeyron equation (*dσ/dT* = −Δ*S*/Δ*ε*). Here, Δ*S* (=*S*_R_ − *S*_B2_ < 0) is the entropy change, and Δ*ε* is transformation strain. The sign of Δ*ε* is positive for V2 and is negative for V1 of the R-phase; therefore, the slope (*dσ/dT*) is positive for V2 and negative for V1. We consider the following two cases: with negligible internal stress field; and with an internal stress field exerted by the aligned coherent particles of Ti_3_Ni_4_.

In the case with negligible internal stress field, there is no difference in transformation temperature between V1 and V2 of the R-phase unless the external stress is applied. As the external stress increases, the B2-R(V2) transformation temperature increases while the B2 → R(V1) transformation temperature decreases, as shown in Fig. 1(a). The B2 → R(V1) transformation, however, cannot be detected experimentally because V2 is more stable than V1 under stress. In this case, the stress-induced B2-R(V2) transformation occurs as indicated by the arrow in Fig. 1(a), while the stress-induced reverse transformation is impossible.

In the case with a strong internal stress field exerted by aligned coherent particles of Ti_3_Ni_4_, the B2-R transformation temperature of V1 is higher than that of V2 because the internal stress selects V1 of the R-phase. This situation is illustrated in Fig. 1(b), where the slope of the phase boundary is assumed to be not influenced by the internal stress. In this case, a stress-induced R(V1) → B2 transformation is possible, as indicated by the arrow in Fig. 1(b). With a further increase in external stress, the B2-phase transforms to the R-phase of the different variant (V2). A thermodynamic explanation of the behavior is given in^19^.

Although the stress-induced R-B2 transformation is detected by the change in resistivity^19^ and temperature change of the specimen^17^, details of the R → B2 transformation is yet to be examined. The present study was motivated to further understand the stress-induced R-B2 transformation by using *in situ* infrared camera and X-ray diffraction. The former method provides information about the temperature distribution of samples and the latter about the changes in crystal structure and the lattice parameters during the transformation.

## Results

### Surface temperature change under tensile stress

The temperature change of the Age300MPa specimen was firstly detected by using a thermocouple attached on the surface of the specimen^17^. To observe the spatially resolved temperature variation, the surface temperatures of the Age300MPa and the Age0MPa specimens were monitored by an IR camera.

**Figure 2:**
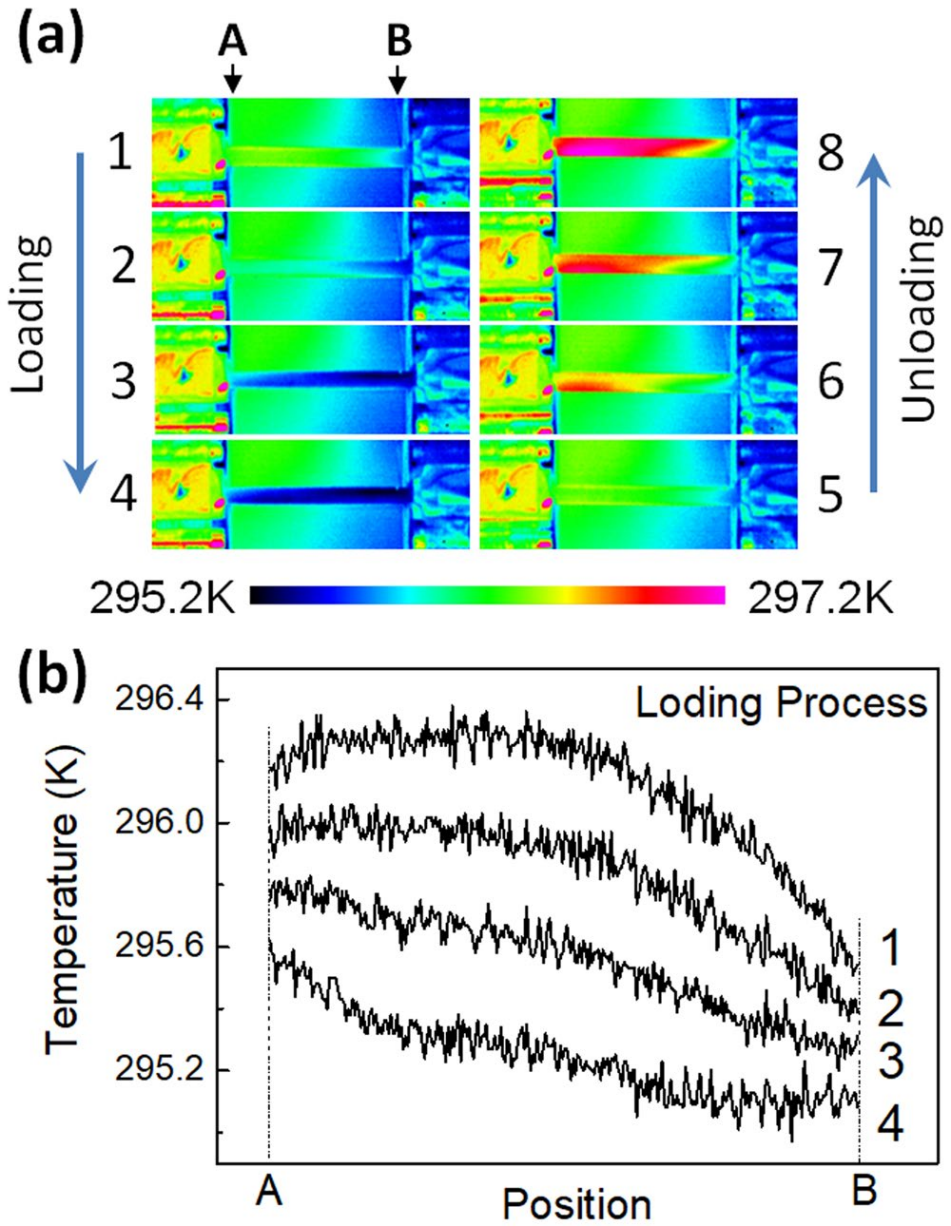
Thermal images of the Age300MPa specimen in the loading and the subsequent unloading processes with the maximum tensile stresses of 200 MPa (**a**) and the temperature distribution of the specimen between A and B (**b**).

Figure 2(a) shows a series of thermal images of the Age300MPa specimen in a cycle of tensile stress application and removal. The maximum stress is 200 MPa, and the ambient temperature is 296 K. At this temperature, the specimen is essentially in the R-phase state with a small fraction of residual B2-phase. The time interval between two adjacent images is ~0.7 s, which corresponds to a stress interval of ~70 MPa. We notice that the temperature of the specimen decreases homogenously during the stress-applying process (1 to 4) and increases homogeneously in the stress-removing processes (5 to 8). This implies that the specimen exhibits stress-induced R(V1) → B2 transformation.

The temperature distributions between the chucks (A-B in Fig. 2(a)) for the states 1 to 4 in (a) is plotted in Fig. 2(b). The temperature of the specimen is almost homogeneous near the center. Similar temperature distribution was observed during the stress-removing process. Incidentally, we notice temperature difference between the center of the specimen and in the vicinity of the chuck. This difference is due to heat transfer between the specimen and chuck.

The temperature distribution of the Age300MPa specimen during the stress-applying process is clearly different from that of binary Ti-Ni alloys exhibiting stress-induced B2-B19’ transformation reported so far^20,21^. According to these reports, there is an obvious contrast in the image between the transformed region and the untransformed region. The homogeneous temperature distribution shown in Fig. 2 implies that every transformed region is too small to be detected within the examined resolution.

**Figure 3:**
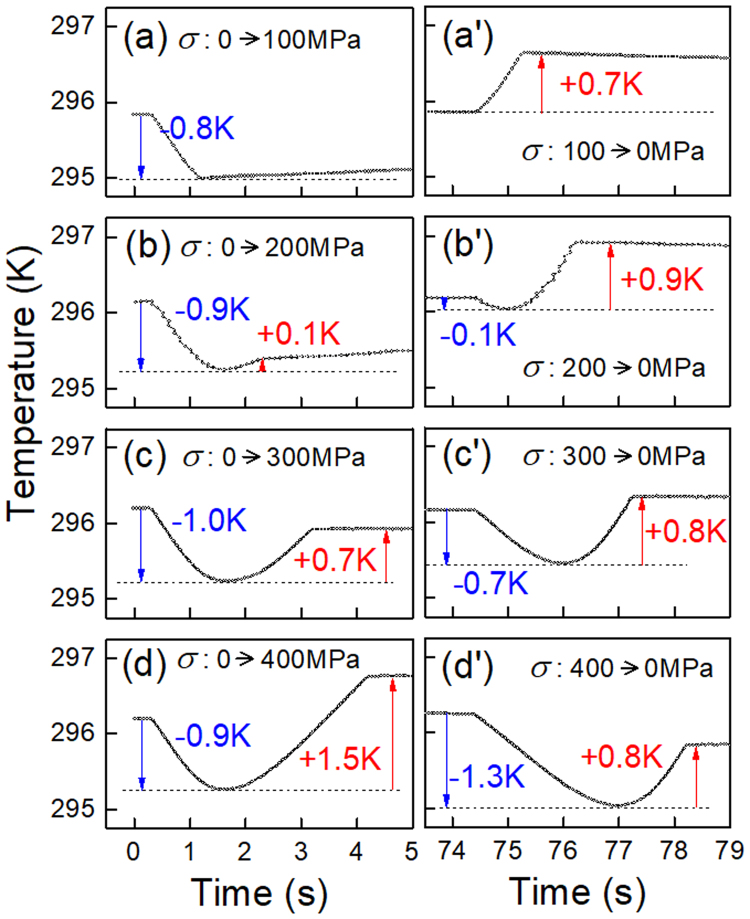
The average temperature change of the Age300MPa specimen during the loading process (**a**–**d**) and the unloading process (a’–d’). The maximum tensile stress is: 100 MPa (a,a’); 200 MPa (b,b’); 300 MPa (c,c’); 400 MPa (d,d’).

The average temperature of the Age300MPa specimen monitored during the stress-applying and the subsequent stress-removing is shown in Fig. 3. When the maximum stress is 100 MPa, the temperature decreases by 0.8 K during the stress-applying process (a) and increases by 0.7 K during the stress-removing process (a’). This is due to the stress-induced R(V1) → B2 transformation as mentioned before. When the maximum applied stress is 200 MPa and higher, the temperature decrease is followed by temperature increase. This means that R(V1) → B2 transformation is followed by the B2 → R(V2) transformation in the stress application process. This behavior corresponds to the vertical arrow shown in Fig. 1(b). In the stress-removing process, the temperature decreases firstly and then increases. This means that R(V2) → B2 transformation is followed by B2 → R(V1) transformation in the stress-removing process. The temperature change caused by the B2 → R(V2) transformation increases as the maximum stress increases. On the other hand, the temperature change caused by the B2-R(V1) transformation is almost independent of stress in the examined stress range, being consistent with the result reported previously^17^.

**Figure 4:**
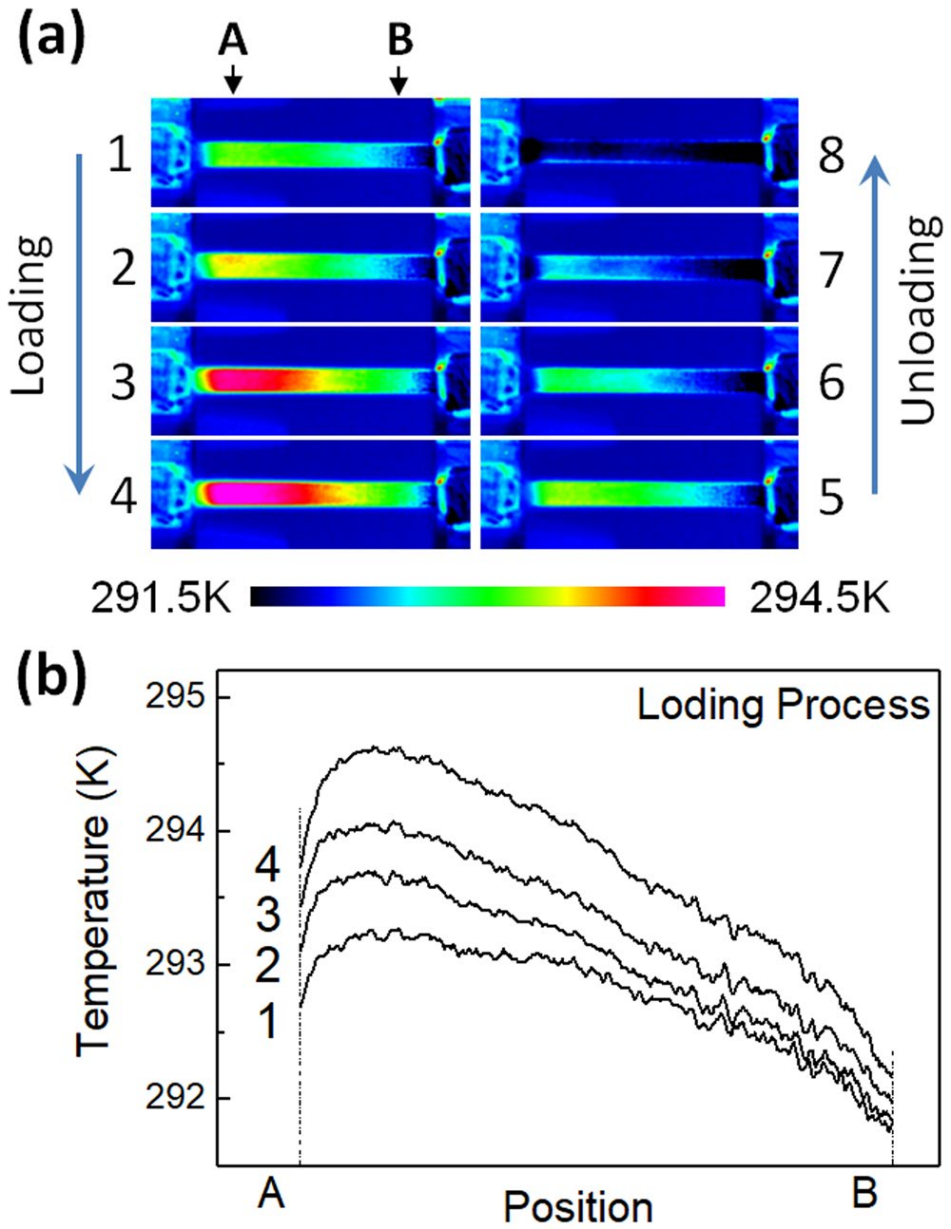
(**a**) Thermal images of the Age0MPa in the loading and the subsequent unloading processes with the maximum tensile stresses of 300 MPa (**a**) and the temperature distribution of the specimen between A and B (**b**).

In the following of this subsection we show the results of the Age0MPa specimen. Figure 4(a) shows thermal images of the Age0MPa specimen in a stress cycle with a maximum stress of 300 MPa. The ambient temperature is 293 K, at which the specimen consists of the B2- and R-phases. The time interval between two adjacent images is 1.0 s, which corresponds to a stress interval of 100 MPa. The temperature of the specimen increases homogeneously during the stress-applying process and decreases homogeneously during the stress-removing process. The temperature distribution between the chucks corresponding to the states 1 to 4 in Fig. 4(a) is shown in Fig. 4(b). The temperature change is again homogeneous thorough the specimen. The homogeneous behavior of the temperature change seems to be a characteristic feature of the aged specimens that include coherent Ti_3_Ni_4_. Similar tests under different applied stresses were also conducted.

**Figure 5:**
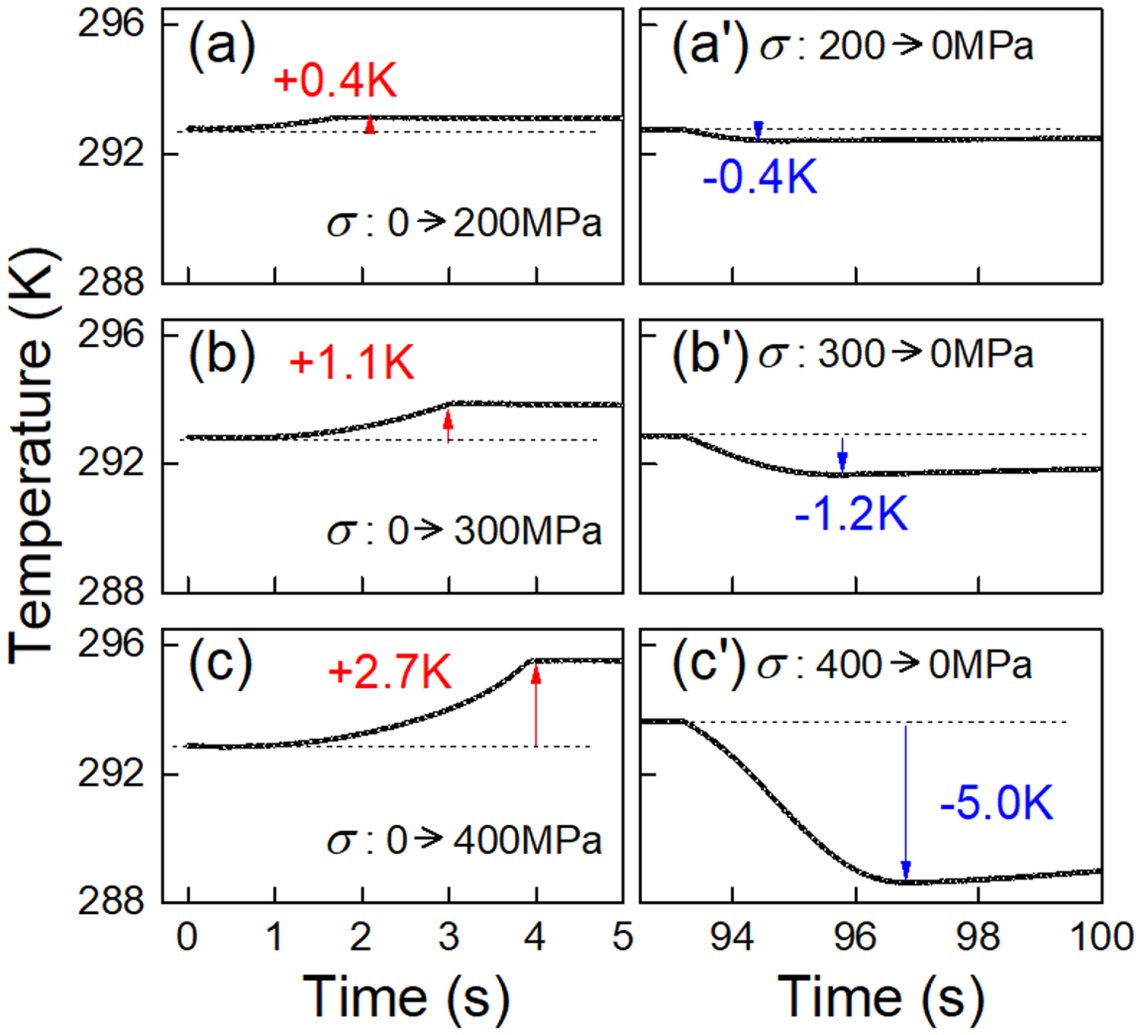
The average temperature change of the Age0MPa specimen during the loading process (**a**–**d**) and the unloading process (a’–d’). The maximum tensile stress is: 200 MPa (a,a’); 300 MPa (b,b’); 400 MPa (c,c’).

The time evolution of the average temperature in the vicinity of the center of the Age0MPa specimen is shown in Fig. 5. Under the maximum applied stress of 200 MPa, the temperature rises by 0.4 K in the stress-applying process, and falls by 0.4 K during the stress-removing process. With increasing the maximum applied stress, the amount of the temperature change continuously increases. The temperature decrease reaches 5 K when the maximum applied stress is 400 MPa. Form the results shown in Figs 4 and 5, we may conclude that stress-induced R(V1) → B2 transformation cannot be detected in the Age0MPa specimen by using infrared camera.

### Structure change under tensile stress

**Figure 6:**
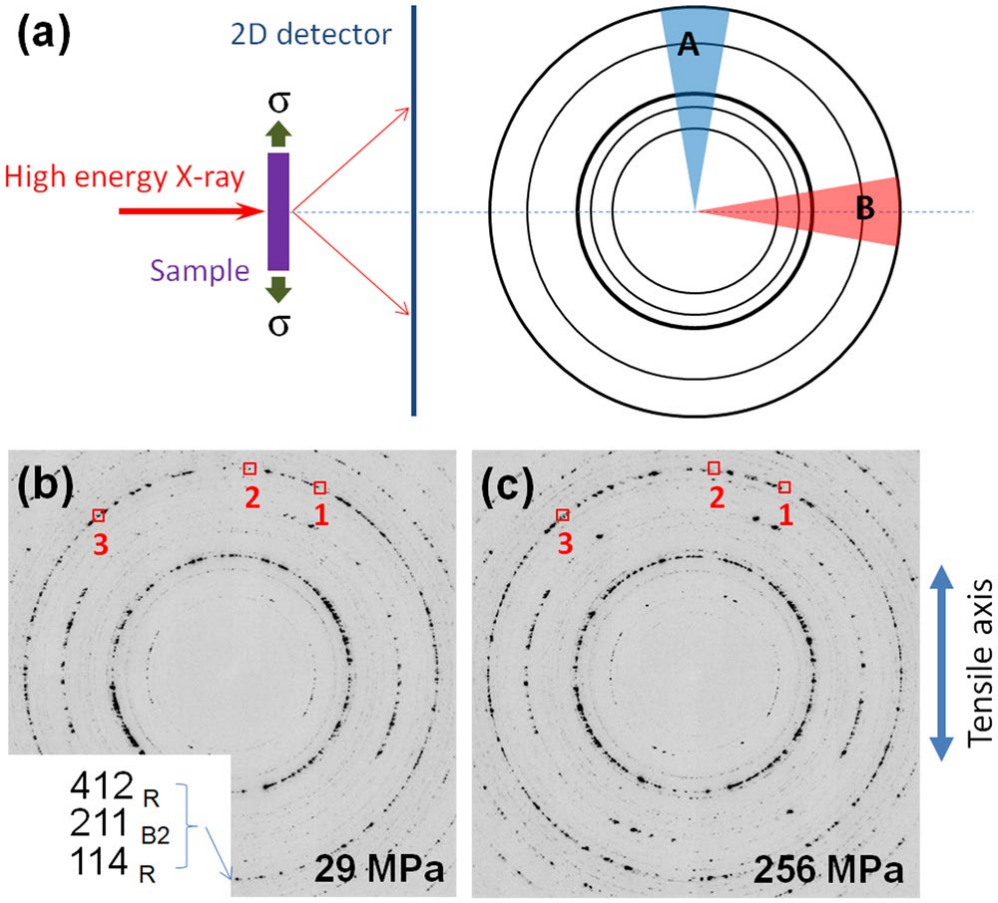
X-ray diffraction pattern of the Age300MPa specimen under tensile stress. Illustration of the set up and Debye ring with two sectors A and B (**a**). Representative diffraction patterns under the tensile stresses of (**b**) 29 MPa and (**c**) 256 MPa. The boxed regions 1, 2 and 3 in (**b**) and (**c**) are further analyzed in Figs 7–9, respectively.

To understand the structure change of the Age300MPa specimen caused by the application of a tensile stress, we performed *in situ* HE-XRD. The geometry of the experiment is illustrated in Fig. 6(a). The incident beam is perpendicular to the tensile direction, and the 2D detector is placed with its normal direction being parallel to the beam direction. The test temperature was 295 K, which is between *M*_s_ and *M*_f_ values of the B2-R transformation (*M*_f_ = 279 K, *M*_s_ = 315 K^17^).

Figure 6(b,c) show representative 2D XRD diffraction patterns obtained under the tensile stresses of 29 MPa and 256 MPa, respectively. These diffraction patterns are essentially composed of the B2-phase, R-phase and Ti_3_Ni_4_. The index of a part of reflections is given in the figure. (The entire index is given in the integrated 1D pattern shown in Fig. s2).

In order to directly confirm stress-induced reverse R(V1) → B2 transformation, detailed analysis was made for the three boxed regions 1, 2 and 3 in Fig. 6(b,c) during the loading process.

**Figure 7:**
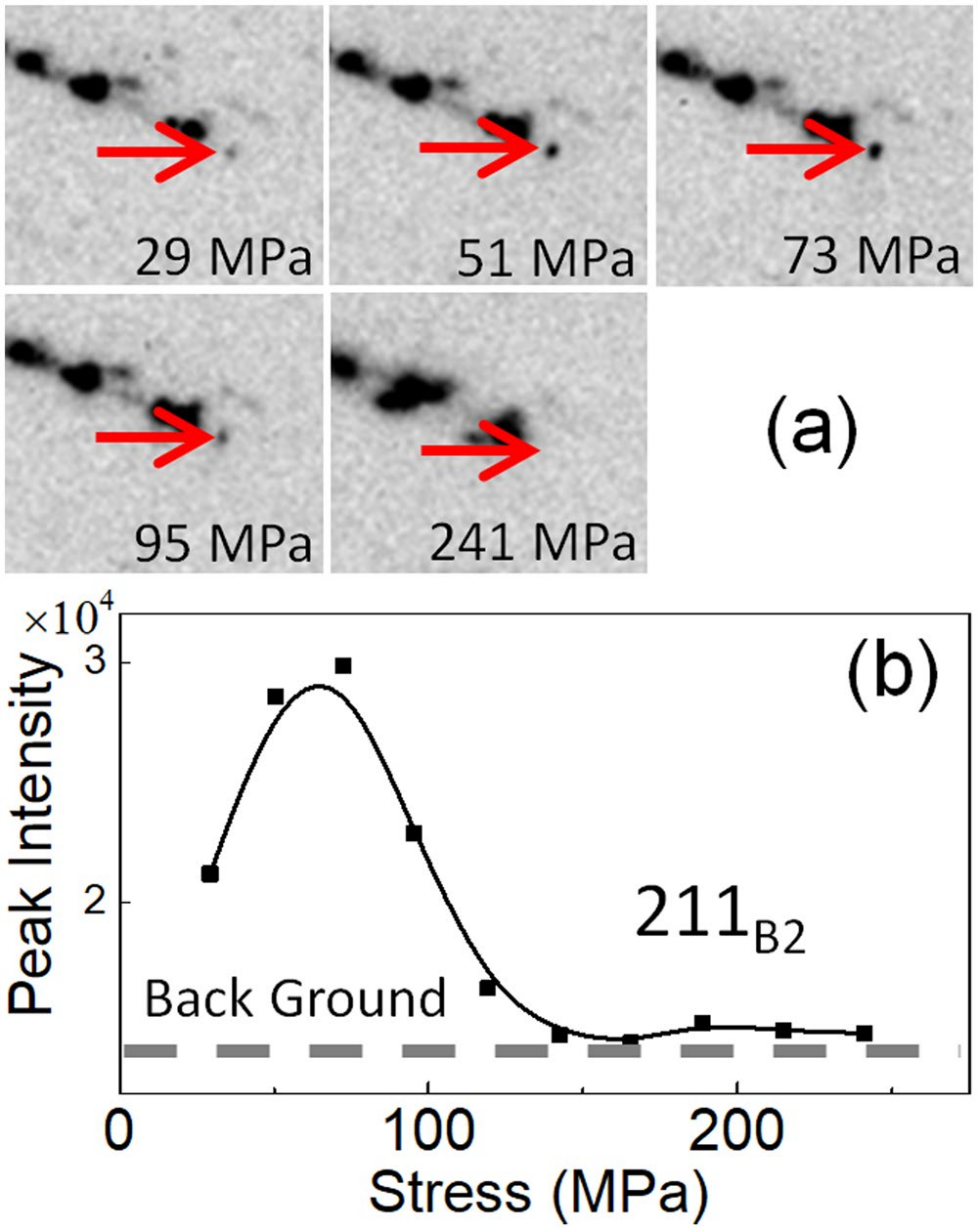
A series of magnified diffraction patterns for the boxed region 1 in Fig. 6 during the loading process (**a**) and the intensity of the 211_B2_ reflection (indicated by an arrow) plotted as a function of applied stress (**b**).

**Figure 8:**
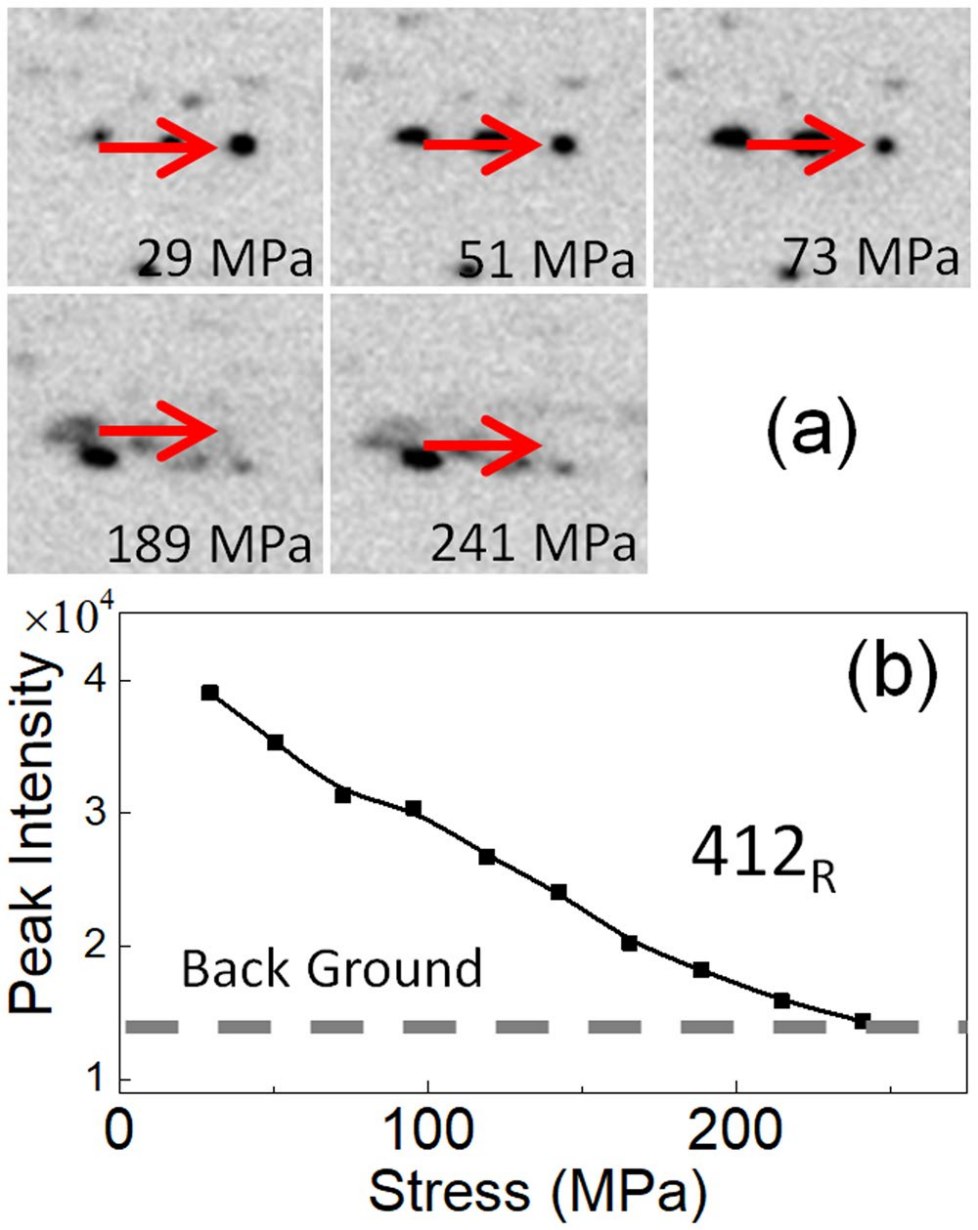
A series of magnified diffraction patterns for the boxed region 2 in Fig. 6 during the loading process (**a**) and the intensity of the 412 _R_ reflection (indicated by an arrow) plotted as a function of applied stress (**b**).

**Figure 9:**
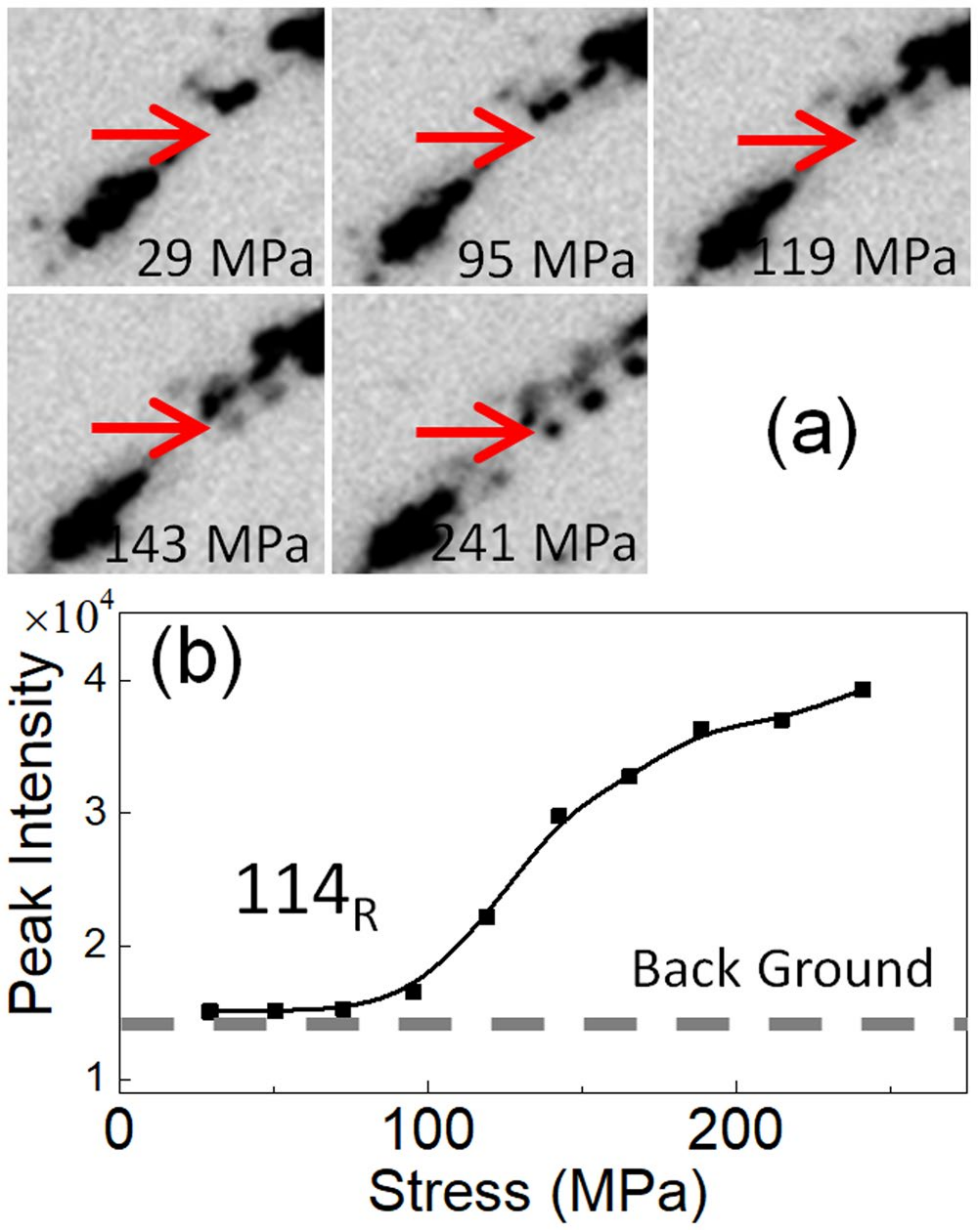
A series of magnified diffraction patterns for the boxed region 3 in Fig. 6 during the loading process (**a**) and the intensity of the 114 _R_ reflection (indicated by an arrow) plotted as a function of applied stress (**b**).

Figures 7, 8 and 9 show the change in the diffraction pattern in the loading process for the regions 1, 2, 3, respectively. The reflections indicated by the arrow in Figs 7(a), 8(a) and 9(a) are 211_B2_, 412 _R_, and 114 _R_, respectively. The 211_B2_ reflection separates into and 412 _R_ and 114 _R_ reflections depending on variants by the B2 → R transformation. From the geometry of the present experiment, the variant of the R-phase contributing to the 412 _R_ reflection in Fig. 8(a) is contracted in the tensile direction while that contributing the 114 _R_ reflection in Fig. 9(a) is expanded in the tensile direction. Therefore, we regard that the 412 _R_ reflection comes from variant V1 and the 114 _R_ reflection comes from variant V2 of the R-phase.

The intensity of the 211_B2_ reflection firstly increases with increasing applied stress then it decreases on further increasing the applied stress as shown in Fig. 7. The increase of the intensity implies the progress of the stress-induced R(V1) → B2 transformation, and the subsequent decrease implies the stress-induced B2-R(V2) transformation. This behavior corresponds to the vertical arrow shown in Fig. 1(b) and is consistent with the temperature change shown in Fig. 3(b). The intensity of the 412 _R_ reflection monotonically decreases as stress increases as shown in Fig. 8. This result also support that the present specimen exhibits stress-induced R(V1) → B2 transformation. The intensity of the 114 _R_ reflection is nearly constant below 100 MPa, and it monotonically increases as stress increases as shown in Fig. 9. This result supports stress-induced B2 → R(V2) transformation.

For further confirmation of stress-induced R(V1)-B2-R(V2) transformation, microstructure observation under tensile stress will be effective and it is a subject in the future.

## Discussion

We first discuss correspondence between the change in temperature and diffraction pattern by the application of tensile stress in the Age300MPa specimen. We observed that the temperature of the specimen initially decrease and then increase in Fig. 3. The initial temperature decrease should be caused by the R(V1) → B2 transformation. This behavior is consistent with the initial increase in the intensity of the 211_B2_ reflection in Fig. 7(b) and the decrease in the intensity of the 412 _R_ reflection in Fig. 8(b). The subsequent temperature increase should be caused by the B2 → R(V2) transformation. This behavior is consistent with the decrease in the intensity of the 211_B2_ reflection above 73 MPa (Fig. 7(b)) and the increase in the intensity of the 114 _R_ reflection (Fig. 9(b)). From these comparison, we conclude that the temperature change of the Age300MPa specimen essentially arises from the structure change of the specimen.

In addition to the structure change (R → B2 and B2 → R transformations) the Aged300MPa specimen also show rearrangement of variants by the application of the stress. This behavior can be explained by comparing Figs 7(b), 8(b) and 9(b). The intensity of the 211_B2_ reflection is nearly zero above 150 MPa. This implies the B2-phase is absent above 150 MPa. However, the intensity of the 412 _R_ reflection decreases and that of the 114 _R_ reflection increases above 150 MPa. This behavior implies that rearrangement of R-phase variant occurs in the specimen by the stress application in addition to stress-induced martensitic transformations.

**Figure 10:**
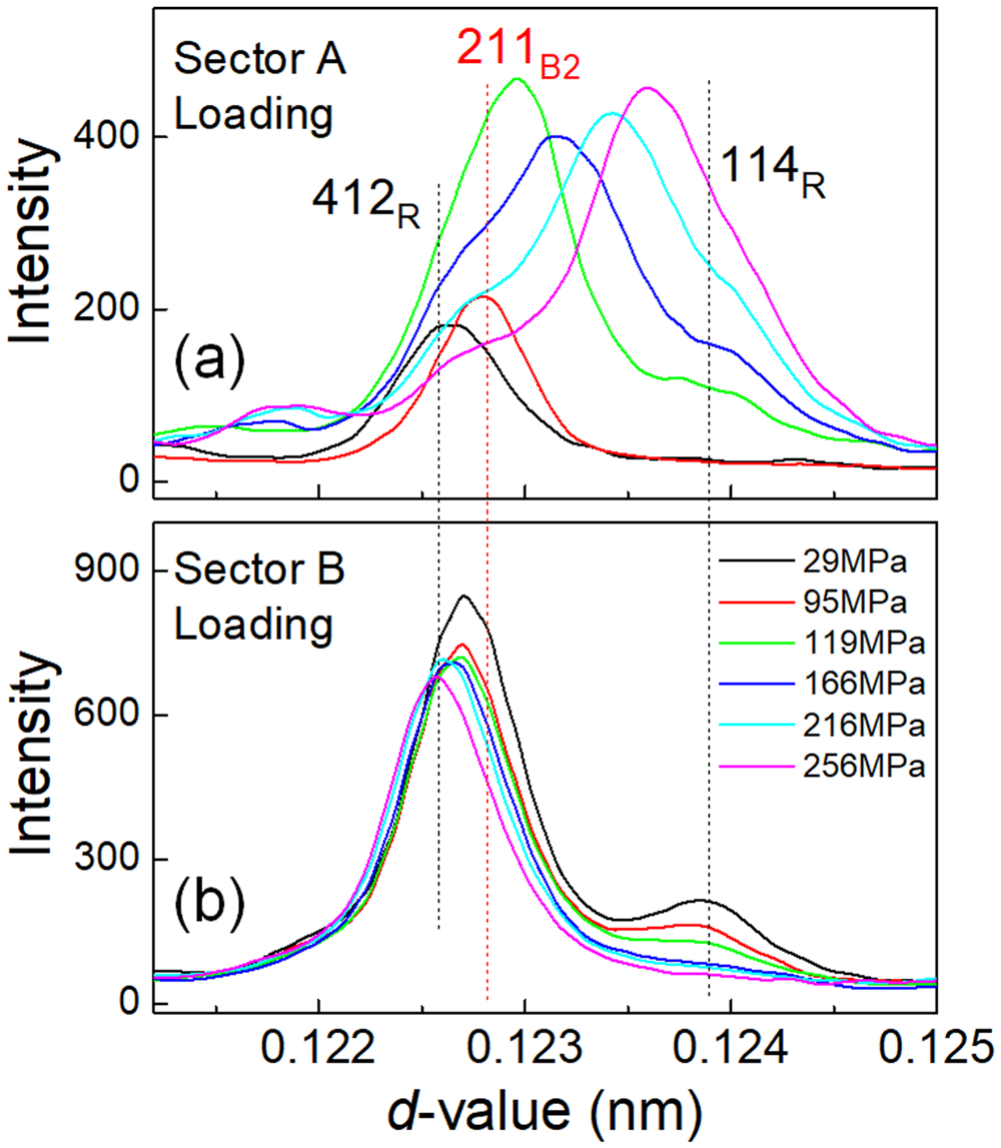
Integrated diffraction patterns for the Ti-51Ni (at%) Age300MPa specimen for the sectors A (**a**) and B (**b**) during the loading process.

To see the change of the diffraction pattern from different aspects, we integrated the 2D pattern within the two sectors indicated by A and B in Fig. 6(a). The angle of these sectors is 20 degrees. The scattering vectors of the reflections within the sector A are close to the loading direction, while those within the sector B are nearly perpendicular to the loading direction. The integrated diffraction patterns in the loading process are presented in Fig. 10(a,b) for the sectors A and B, respectively. These diffraction patterns are essentially composed of the 412 _R_ and 114 _R_ reflections. We may expect the existence of 211_B2_ reflection at the position indicated by the dotted red line, but it is almost immersed in the tails of the 412 _R_ reflection.

From the geometry of the present experiment we can corresponds these reflections with the variants of the R-phase. The 412 _R_ reflection in sector A comes from V1 and that in sector B from V2 because the *d*-value of 412 _R_ is smaller than that of 211_B2_. The 114 _R_ reflection in sector A comes from V2 and that in sector B from V1 because the *d*-value of114_R_ is larger than 211_B2_.

In sector A (Fig. 10(a)), the intensity of the 412 _R_ reflection decreases and that of 114 _R_ reflection increases by the stress application. The behavior is consistent with the results shown in Figs 8(b) and 9(b). In sector B (Fig. 10(b)), on the contrary, the intensity of the 412 _R_ reflection is almost constant while that of 114 _R_ reflection decrease as stress increases. The decrease in the 114 _R_ reflection in sector B is due to the stress induced R(V1) → B2 transformation or rearrangement of variants (V1 → V2). For both sectors, it is difficult to extract the change in the intensity of the 211_B2_ reflection.

**Figure 11:**
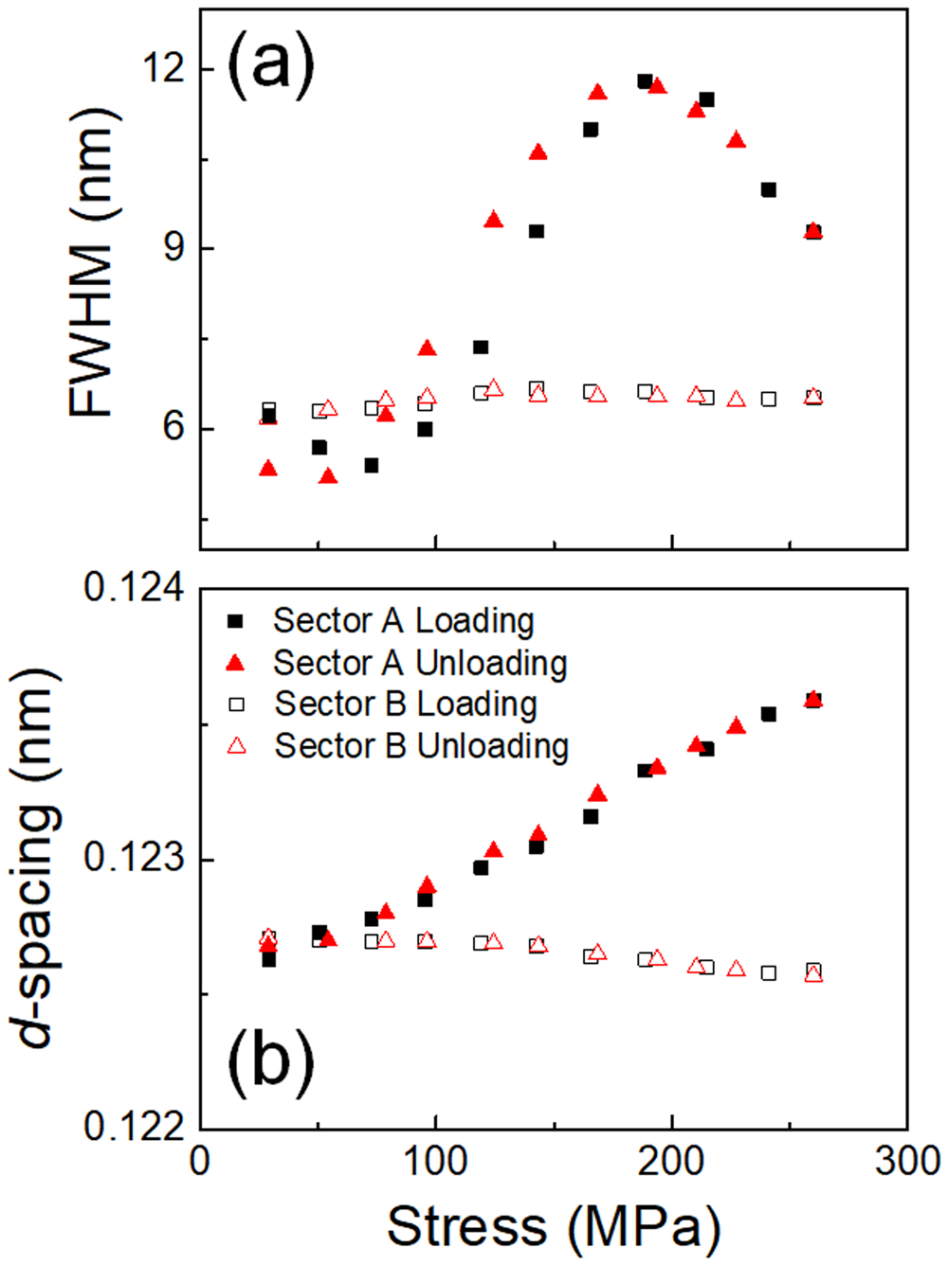
Stress dependencies of FWHM (**a**) of the diffraction peaks and *d*-values (**b**) during the loading and unloading processes for the sectors A and B of Age300MPa specimen.

To understand the change in profile we approximate each profile shown in Fig. 10(a) as a single peak containing shoulders. We also approximate each profile shown in Fig. 10(b) as a single peak with an additional small peak of 114 _R_. Then we evaluate the full width at half maximum (FWHM) value and the *d*-value of the approximated single peak and the result is shown in Fig. 11(a,b), respectively. The FWHM of sector A first increases and then decreases as the applied stress increases. This increase is probably due to the increase in the intensity of the 211_B2_ reflection, which is caused by the stress-induced R(V1)-B2 transformation. The subsequent decrease in FWHM value is due to the decrease in the intensity of the 211_B2_ reflection, which is caused by the stress-induced B2-R(V2) transformation. On the other hand, the FWHM of sector B keeps almost constant.

The *d*-value evaluated from sector A monotonically increases while that from sector B monotonically decreases as the stress increases. The slope of the *d*-value (from sector A) vs stress curve corresponds to the inverse of the Young’s modulus. The Young’s modulus is approximately 37 GPa when the applied stress is below 100 MPa, where stress-induced R-B2 transformation is in progress, while the value is approximately 27 GPa when the applied stress is between 100 MPa and 200 MPa, where the stress-induced B2-R transformation is in progress.

In the following section, we discuss the change in the integrated X-ray profile caused by stress-induced R(V1)-B2-R(V2) transformation. The diffraction pattern shown in Fig. 10 is a mixture of V1 and V2 of the R-phase and the B2-phase. The gradual movement of the reflection from 412 _R_ to 114 _R_, as seen in Fig. 10(a), occurs because of two events: one is a gradual change in the lattice parameter due to elastic deformation, and the other is a stress-induced successive R(V1)-B2-R(V2) transformation.

As the two events occur simultaneously, it is difficult to see the stress dependence of *d*-values of each phase separately. The change in *d*-values by stress application also makes it difficult to observe a specific structure change caused by stress-induced martensitic transformation in the relative large integrated sectors. Nevertheless, stress dependence of each reflection in the 2D diffraction patterns (Figs 7–9) clearly shows the stress-induced R(V1)-B2-R(V2) transformation.

The reason for the stress-induced reverse R-B2 transformation is mainly attributed to the existence of an internal stress and the difference in the stress dependence of the transformation temperature between the different variants as shown in Fig. 1. In the case of the present Ti-51Ni alloy, aligned coherent particles of Ti_3_Ni_4_ precipitate is the main cause of internal stress. It has recently been reported that a ribbon of a Ni-Mn-Sn alloy, which does not include coherent precipitates, also shows a stress-induced reverse martensitic transformation due to an internal stress formed in the ribbon through its production^18^. This implies that stress-induced reverse transformation is not a unique case. These transformations may occur in many alloys in which strong internal stresses are formed.

Concerning the difference in stress dependence of the transformation temperature between different variants shown in Fig. 1, we would like to mention a similar behavior in the magnetic field dependence of the transformation temperature of Ni_2_MnGa^22^. The martensite phase of Ni_2_MnGa has strong magnetocrystalline anisotropy. Therefore, a large difference in magnetic energy arises between different variants when a magnetic field is applied. Thus, the magnetic field dependence of the transformation temperature is different between variants: the transformation temperature increases with increasing magnetic field when the magnetic field is parallel to the easy axis while it decreases when the magnetic field is perpendicular to the easy axis^22^.

## Conclusions

In summary, stress-induced reversible transformations in the Ti-51Ni (at%) alloy containing aligned coherent particles of Ti_3_Ni_4_ were investigated using an infrared camera and *in situ* X-ray diffraction. We found that the stress-induced R(V1)-B2-R(V2) transformation proceeds with a homogeneous change in temperature within the resolution of the infrared camera. In addition, we confirmed that the stress-induced reverse R-B2 transformations appear as changes in the 2D X-ray diffraction profiles. In the tensile direction, the intensity of the 412 _R_ reflection decreases and that of the 114 _R_ reflection increases; the intensity of the 211_B2_ reflection firstly increases then decreases in the loading process. We have also proposed a stress-temperature phase diagram for the interpretation of stress-induced R(V1)-B2-R(V2) transformations.

## Methods

### Sample preparation and characterization

An ingot of Ti-51Ni (at%) alloy was prepared by induction melting and was cast into an iron mold. It was hot rolled into a 1.3 mm thick plate. Tensile test specimens with dimensions of 30 mm in gauge length, 3 mm in width and 0.6 mm in thickness were cut from the rolled plate so that the tensile axis was oriented in the rolling direction. The specimens were solution-treated at 1123 K for 3.6 ks followed by quenching into ice water, then aged at 773 K for 6 ks under an external tensile stress of 300 MPa applied parallel to the tensile axis of the following experiments. We call these specimens Age300MPa. An electron backscattering diffraction analysis revealed that the Age300MPa specimens have a weak (102) <010> texture with an average grain size of 140 μm (Fig. s1 in supplement). For comparison, specimens aged without external stress were also prepared. We call these specimens Age0MPa. The martensitic transformation temperatures are *M*_s_ (R) = 315 K, *A*_f_ (R) = 320 K, *M*_s_ (B19’) = 221 K, and *A*_f_ (B19’) = 297 K for the Age300MPa specimen^17^; *M*_s_ (R) = 302 K, *A*_f_ (R) = 307 K, *M*_s_ (B19’) = 214 K, and *A*_f_ (B19’) = 290 K for Age0MPa specimen^23^. These values were obtained by differential scanning calorimetry.

### Measurements of elastocaloric effect

The temperature variations during stress applying and removing processes were measured using a stress-controlled dynamic testing machine (BOSE 3510) at different temperatures in a stress range of 4–400 MPa with a stress rate of 100 MPa/s. This stress rate is employed to approximate the adiabatic condition. One side of the specimen was coated with a thin black paint, and the temperature of this side was monitored using a multi-detector infrared (IR) camera FLIR SC7700M at a frequency of 50 Hz.

### High energy X-ray scattering

*In situ* high energy (photon energy = 105.7 keV) X-ray diffraction (HE-XRD) experiments under tensile stresses were performed at the beam line 11-ID-C of Advanced Photon Source, Argonne National Laboratory. A beam with a wavelength of 0.01173 nm was irradiated perpendicular to the tensile direction. A PerkinElmer *α*-Si flat-panel large-area detector was used to collect two-dimensional (2D) diffraction patterns. At each stress state, the sample was oscillated ±5° about the tensile axis. The software package FIT2D was used for the diffraction data analysis.

## Electronic supplementary material

Supplementary Dataset 1
