# Supplementary material for: Stress-induced reverse martensitic transformation in a Ti-51Ni (at%) alloy aged under uniaxial stress
Source: Sci Rep. 2018 Apr 17;8:6099. doi: 10.1038/s41598-018-24411-1 (PMC5904179; doi:10.1038/s41598-018-24411-1)
Supplement: Supplementary file 1 — Supplementary Dataset 1 [file 41598_2018_24411_MOESM1_ESM.docx]

**Stress-induced reverse martensitic transformation in a Ti-51Ni (at%) alloy aged under uniaxial stress**

Fei Xiao^1,4^, Hong Chen^1^, Xuejun Jin^1,2^, Zhihua Nie^3^, Tomoyuki Kakeshita^4^ and Takashi Fukuda^4^

*^1^State Key Lab of Metal Matrix Composite*, *School of Materials Science and Engineering, Shanghai Jiao Tong University, 800 Dong Chuan Road, Shanghai 200240, P. R. China*

*^2^Institute of Advanced Steels and Materials, School of Materials Science and Engineering, Shanghai Jiao Tong University, Shanghai 200240, People’s Republic of China*

*^3^School of Materials Science and Engineering, Beijing Institute of Technology, Beijing 100081, China*

*^4^Department of Materials Science and Engineering, Graduate School of Engineering, Osaka University, 2-1, Yamada-oka, Suita, Osaka 565-0871, Japan*

Correspondence and requests for materials should be addressed to X.J. (email: jin@sjtu.edu.cn) or Z.N. (email: znie@bit.edu.cn) or T.F. (email: fukuda@mat.eng.osaka-u.ac.jp)


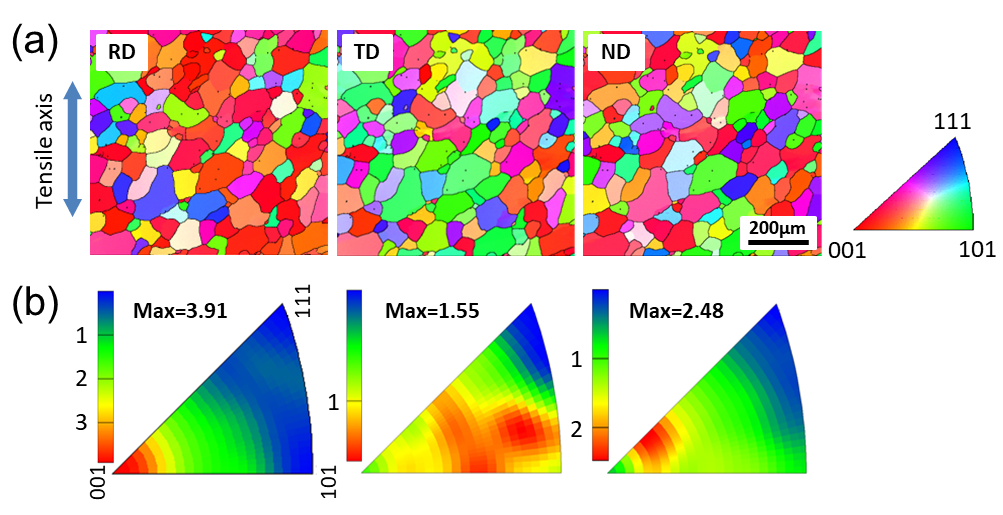


Figure s1. (a) A quasi-colored orientation mapping of microstructures and (b) inverse pole figures by EBSD for the Ti-51Ni (at%) Age300MPa specimen at room temperature. RD, TD and ND are rolling, transverse and normal directions, respectively. The tensile direction is parallel to RD. This sheet has a weak (1 0 2)<0 1 0> recrystallization texture, where the texture intensity of the <010>_B2_ component in the RD is 3.91. In addition, there are some more weak recrystallization textures, such as (3 0 17)<17 10 3>, (101)<414> and (118)<081>. In the (102)<010> textured sheet, the <010>_B2_ direction, which is parallel to the RD is as tensile direction.


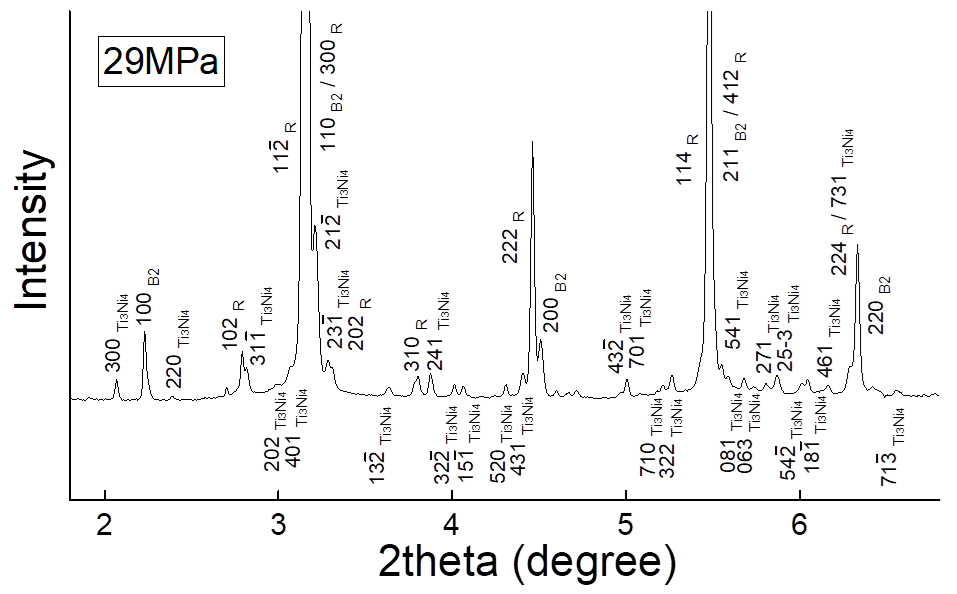


Figure s2. The entire index of the integrated 1D pattern for the Ti-51Ni (at%) Age300MPa specimen under a constant tensile stress of 29 MPa at room temperature.
